# Reflex control of ipsilateral and contralateral paraspinal muscles

**DOI:** 10.1007/s00221-012-3032-9

**Published:** 2012-02-19

**Authors:** Iain D. Beith

**Affiliations:** School of Rehabilitation Sciences, Joint Faculty of Health and Social Care Sciences, Kingston University/St Georges University of London, London, UK

**Keywords:** Stretch reflex, Paraspinal muscles, Crossed reflex

## Abstract

Homonymous and heteronymous reflex connections of the paraspinal muscles were investigated by the application of a tap to the muscle bellies of the lumbar multifidus and iliocostalis lumborum muscles and observation of surface electromyographic responses in the same muscles on both sides of the trunk. Reflexes were evoked in each of the homonymous muscles with latencies and estimated conduction velocities compatible with being evoked by Ia muscle afferents and having a monosynaptic component. Short latency heteronymous excitatory reflex connections were observed in muscles on the ipsilateral side, whilst reflex responses in the contralateral muscles were inhibitory in response to the same stimulus. The latencies of the crossed responses were on average 9.1 ms longer than the ipsilateral excitatory responses. These results are in contrast to the crossed excitatory responses observed between the abdominal muscles and trapezius muscles on the opposite aspect of the trunk. Such a difference in the reflex pathways between these two groups of trunk muscles compliments the different anatomical arrangement of the muscle groups and suggests a contribution to their commonly observed activation patterns.

## Introduction

It is widely presumed that stretch reflex activity in postural muscles is relatively potent and widespread, and this has been confirmed by investigating the latency amplitude and interconnectivity in stretch reflexes of the abdominal muscles (Myriknas et al. 2000; Beith and Harrison 2004). The homonymous reflexes evoked in these muscles have latencies consistent with Ia muscle afferents, are relatively large, and the heteronymous responses identified are also large and excitatory even when they cross the spinal cord. In addition, similar though smaller crossed excitatory reflex responses have been observed between the trapezius muscle on ipsilateral and contralateral sides (Alexander and Harrison 2002).

Such crossed reflex pathways compliment the commonly observed co-activation of these muscles on either side of the trunk during everyday activities (Peach et al. 1998; Alexander and Harrison 2002) and are therefore likely to contribute to the control of these muscles during these tasks. In addition, such reflex connections across the spinal cord seem to compliment the anatomical connections of the left and right abdominal muscles that have a common central tendon (Askar 1977; Rizk 1980) and the left and right trapezius muscles that attach to either side of each thoracic spinous process (Standring et al. 2004). These connections ensure the muscles on left and right sides are connected and in series. Such neural and mechanical connectivity seems likely to aid the maintenance of posture and stability of the trunk and the spine.

Whether such reflex pathways exist between the paraspinal muscles on the extensor aspect of the trunk and whether they compliment known activation patterns of the muscles on either side of the trunk is still unclear. The aim of the present study was, therefore, to investigate the reflex connectivity of the paraspinal muscles by the application of a tap to individual muscles and the observation of reflexes both homonymously and heteronymously on ipsilateral and contralateral sides of the trunk.

## Methods

Short latency reflex connections between the lumbar multifidus and iliocostalis lumborum muscles were investigated in 18 subjects (5 men, 13 women), with an average age of 32 ± 7 years (mean ± SD). Subjects were excluded if they had any movement or neurological disorders, or current low back pain. All procedures conformed to the standards set by the Declaration of Helsinki were approved by the local research ethics committee of King’s College, London, and informed written consent was obtained from each subject.

**Fig. 1:**
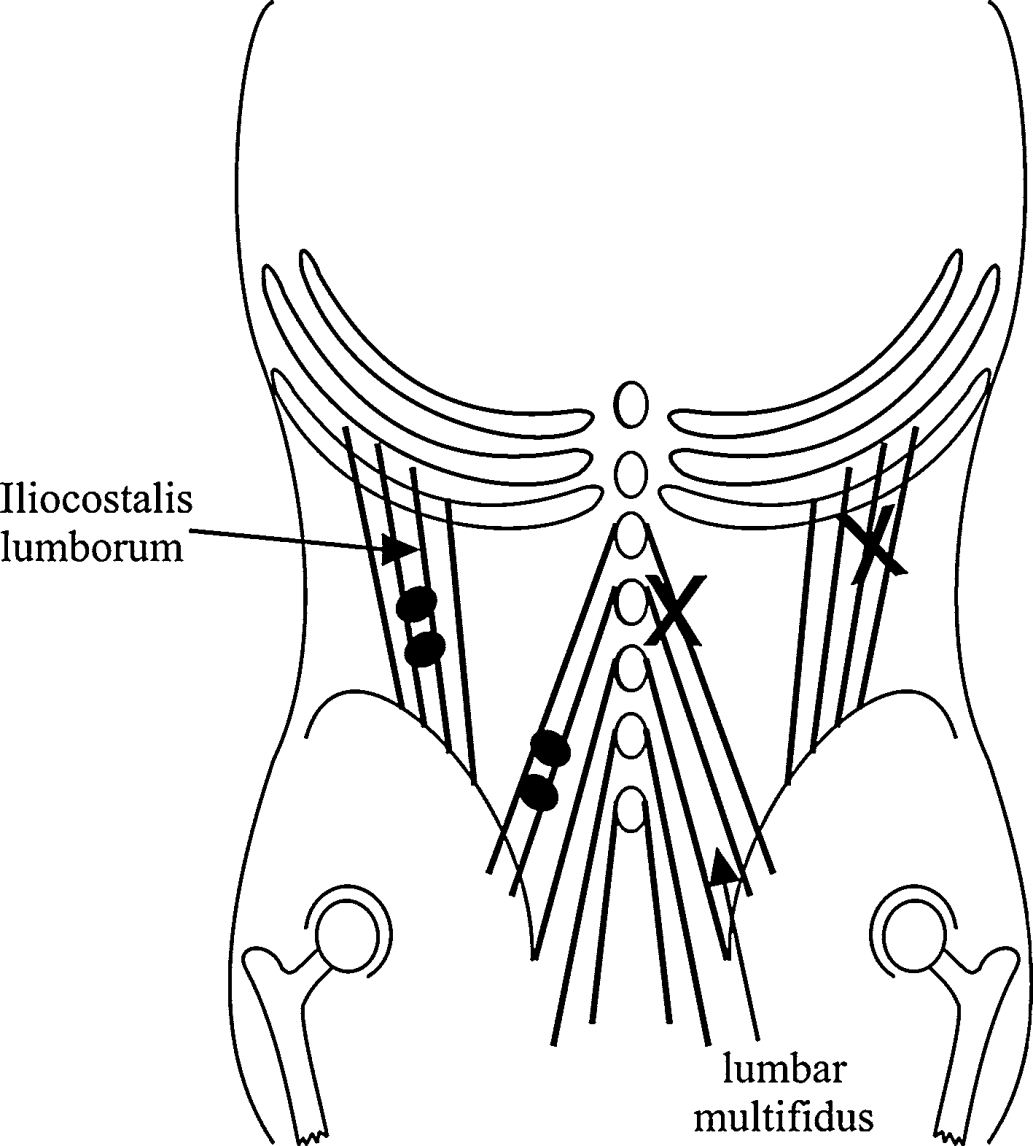
Paraspinal muscles tapped and recorded from. The points at which the taps were applied are indicated by crosses, and the position of the surface electrodes to detect EMG by pairs of *black circles*. Taps were applied at the lateral cross to stretch the iliocostalis lumborum muscle (iliocostalis lumborum), and at the cross near the midline to stretch the lumbar multifidus muscle (lumbar multifidus). The laterally placed electrode detects activity from iliocostalis lumborum and those nearer the *midline* to detect activity from lumbar multifidus. For ease of presentation, the points tapped and the electrodes are positioned on one side only, but for all experiments, both sides were tapped and recorded from

All experiments were undertaken by the same investigator whilst subjects were standing symmetrically and leaning slightly forward to raise the background level of EMG in the paraspinal muscles. Whilst levels of EMG activity were not measured, such consistent symmetry and minimal forward lean ensured consistent task performance across subjects and therefore comparable levels of EMG activity between subjects. Reflex activity was induced by means of a mechanical tap. This was produced by a tapping device consisting of a drive unit to which was attached a round polyethylene cap 10 mm in diameter, which was then used as a probe to apply taps to the proximal end of the muscle bellies of both left and right lumbar multifidus and iliocostalis lumborum muscles (Fig. 1). The tapper was driven by a 1-ms square wave pulse. The amplitude of the resultant tap measured when the probe was driven without a load was 1.9 mm with a time to peak amplitude of 1.9 ms. (No measure of amplitude when loaded against tissue was possible, but this may have been as little as 0.19 mm). These measures would give a velocity of 100 mm/s—an effective velocity to activate muscle spindle Ia afferents (Matthews 1972).

When evoking H reflex responses, interstimulus intervals of less than 4 s lead to a depression in the size of the second response due to post-activation depression (Stein et al. 2007; van Boxtel 1986). However, van Boxtel (1986) demonstrated that a short interstimulus interval had less effect on the size of mechanically evoked responses. To test this, preliminary experiments of the tap-evoked response in multifidus and iliocostalis lumborum using interstimulus intervals of 1, 2 and 5 s were conducted. There was no difference in either frequency of occurrence or amplitude of the responses, so the experiments were undertaken using interstimulus intervals of 1 or 2 s.

In preliminary experiments, tapping on or immediately adjacent to the vertebral spinous processes was found to evoke excitatory responses bilaterally as shown by (Dimitrijevic et al. 1980; Tani et al. 1997). It is not clear whether these excitatory responses were evoked due to distortion of both left and right tendons by the tapping action or by evoking responses from ligamentous structures attached to bone. Whichever mechanism evokes such bilateral excitatory responses, the origin of the afferent activity would be less clear blurring identification of the afferent source. In addition, any possible crossed inhibitory responses would not be evident against the excitatory activity evoked. Therefore, to minimise such uncertainty, care was taken to apply the tap to the lumbar multifidus muscle opposite L2 1 cm away from its insertion to the spinous process so as to isolate the stimulus to the muscle spindles within the lumbar multifidus itself (Fig. 1). When doing so, excitatory activity was rarely seen in the contralateral muscles. The iliocostalis lumborum muscle was tapped 1 cm below the upper insertion point on to the ribs (Fig. 1).

The tapping device and the recording equipment were triggered via a digital pulse, generated from Signal software version 1.9 or 2 [Cambridge Electronic Design (CED)]. The EMG was preamplified (Neurolog NL824), isolated and amplified (NL820), (overall gain 2000), band pass filtered between 30 Hz and 3 kHz (NL125), displayed on an oscilloscope, converted into digital format (1401 Micro, CED) and was sampled using Signal software [version 1.9 or 2 (CED)] at 10 kHz and stored on computer for off line analysis. The EMG activity was collected for a period of 200 ms, with 50 ms of data usually being collected before the stimulus and 150 ms of data after the stimulus.

EMG was recorded via self-adhesive surface electrodes (Medicotest blue sensor) positioned with an inter-electrode distance of 2 cm. The electrodes were positioned on the skin over the left and right lumbar multifidus muscle bellies, and the left and right iliocostalis lumborum muscle bellies, in line with the fibre direction of the underlying muscle (Fig. 1; see De Foa et al. 1989). The lumbar multifidus is arranged in segments from deep to superficial and is overlayed by fascia, so the most superficial lumbar multifidus segments are the closest muscle fibres to the overlying electrodes (Macintosh et al. 1986). The muscle is also arranged segmentally from each lumbar vertebra as illustrated in Fig. 1 (Macintosh et al. 1986; Shindo 1995). The point of tap and detecting electrodes were positioned in line over and along the length of the L1 and L2 segments of lumbar multifidus. Tapping 1 cm lateral to L2 localised the stretch produced to the L1 and L2 segments. The electrodes positioned directly over the distal end of the L1 and L2 segments were in parallel with the direction of fibres, to localise the signal detected from the same segments and to optimise the signal detected (Beith and Harrison 2004). A model by Fuglevand et al. (1992) predicts that 90% of EMG signal detected by surface electrodes positioned 2 cm apart is from tissue within 12 mm of the electrodes and that 99% of the signal is dissipated at a distance of 18 mm from the electrodes. Given the anatomy of the lumbar multifidus outlined above, this suggests that the majority of the signal from each pair of electrodes directly over lumbar multifidus will detect electrical activity primarily from that muscle.

### Identification of reflex activity

A reflex was accepted as being present if on the rectified and averaged traces, the level of EMG activity rose above two standard deviations (2SD) from the background EMG (Wood and Smith 1992) for over 5 ms.

### Analysis of data

The latencies of all reflex responses were identified visually from the non-rectified averaged data, as the first clear deflection from the background noise when the data rose above (excitatory) or dipped below (inhibitory) 2SD of the background noise measured in the 50 ms of data collected immediately prior to the stimulus (Beith and Harrison 2004). Such responses were then accepted as being short latency reflexes only when the change in EMG activity was continuous for over 5 ms (Wohlert 1996; Wood and Smith 1992).

Estimations of reflex pathway conduction velocities were calculated from the measures of peripheral nerve pathways taken from human skeletons and compared to reflex latencies as follows. It was assumed that the afferent and efferent conduction velocities are the same for all pathways. The lumbar multifidus is known to be a segmented muscle originating from the five lumbar vertebrae, and that the muscle fibres attaching proximally to a vertebra are supplied by the nerve root exiting from the same vertebral level (Macintosh et al. 1986; Shindo 1995). The distance of the pathway was measured from where the EMG recording electrodes were positioned over the lateral part of lumbar multifidus supplied by L1 to the estimated position of the motoneurone pool of L1 at T9 vertebral level (Carpenter, 1985) on five skeletons as 0.50 ± 0.02 m. Calculation of the conduction velocity was made comparing this distance to the average reflex latency minus the time course of the delay for transmission across the neuromuscular synaptic cleft and the monosynaptic connection onto the motoneuron in the spinal cord. The delay for chemical transmission across the synaptic cleft is approximately 1 ms (Katz and Miledi 1965), so 2 ms was subtracted from the reflex latency before calculating the conduction velocity.

In contrast to lumbar multifidus, neither the course of the nerve supply or the position of the motoneurone pool of the iliocostalis lumborum muscle has been reported in detail. The muscle attaches to the lower ribs and extends to the pelvis and therefore does not attach to different vertebral levels. Whilst the nerve supply is from Lumbar 1 to Lumbar 4, the exact segmental level which supplies each part of the muscle is uncertain and the level of the motoneurone pool supplying the muscle underlying the surface electrodes is unknown. Two assumptions were therefore made. Firstly, that the peripheral nerve entered and exited via the upper lumbar vertebrae, and secondly, that the iliocostalis lumborum motoneurone pool is situated in the upper lumbar spinal cord level with T9. The distance from the position of the recording over the iliocostalis lumborum muscle, to the upper lumbar vertebral column via this course, and then to T9 and back to the muscle, was measured on five skeletons as 0.42 ± 0.01 m. Calculation of the conduction velocity was made as for lumbar multifidus outlined above including subtraction for neuromuscular and synaptic delays.

Reflex amplitude was measured using the peak value of the rectified and averaged data relative to the mean level of rectified and averaged EMG activity immediately prior to the stimulus, as used by Evans et al. (1991), and us (Beith and Harrison 2004). The frequency of occurrence of reflexes was expressed as the number of times a reflex was observed relative to the number of trials and described as a percentage.

## Results

### Homonymous responses in lumbar multifidus and iliocostalis lumborum

**Fig. 2:**
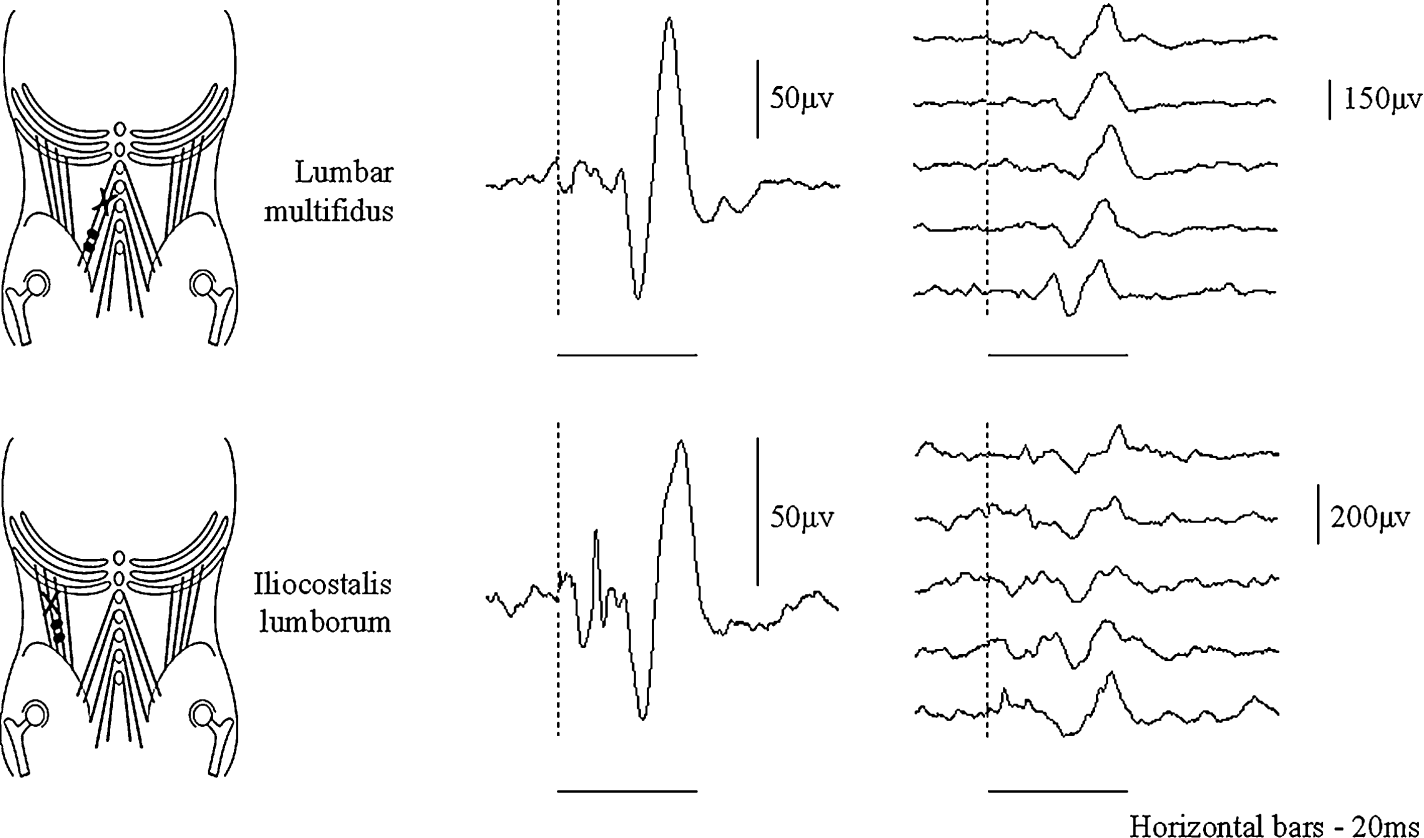
Short latency reflexes in the lumbar multifidus and iliocostalis lumborum. The points tapped on the lumbar multifidus (*upper* illustration) and iliocostalis lumborum (*lower* illustration) are shown with an X and the position of the electrodes as pairs of *black dots*. The *middle* traces are the average of 30 taps, and five consecutive individual sweeps of the thirty, which make up these averages are shown on the right of the respective average. The tap was applied at the point of the *dashed vertical line* on the traces. *Horizontal bar*—20 ms

**Fig. 3:**
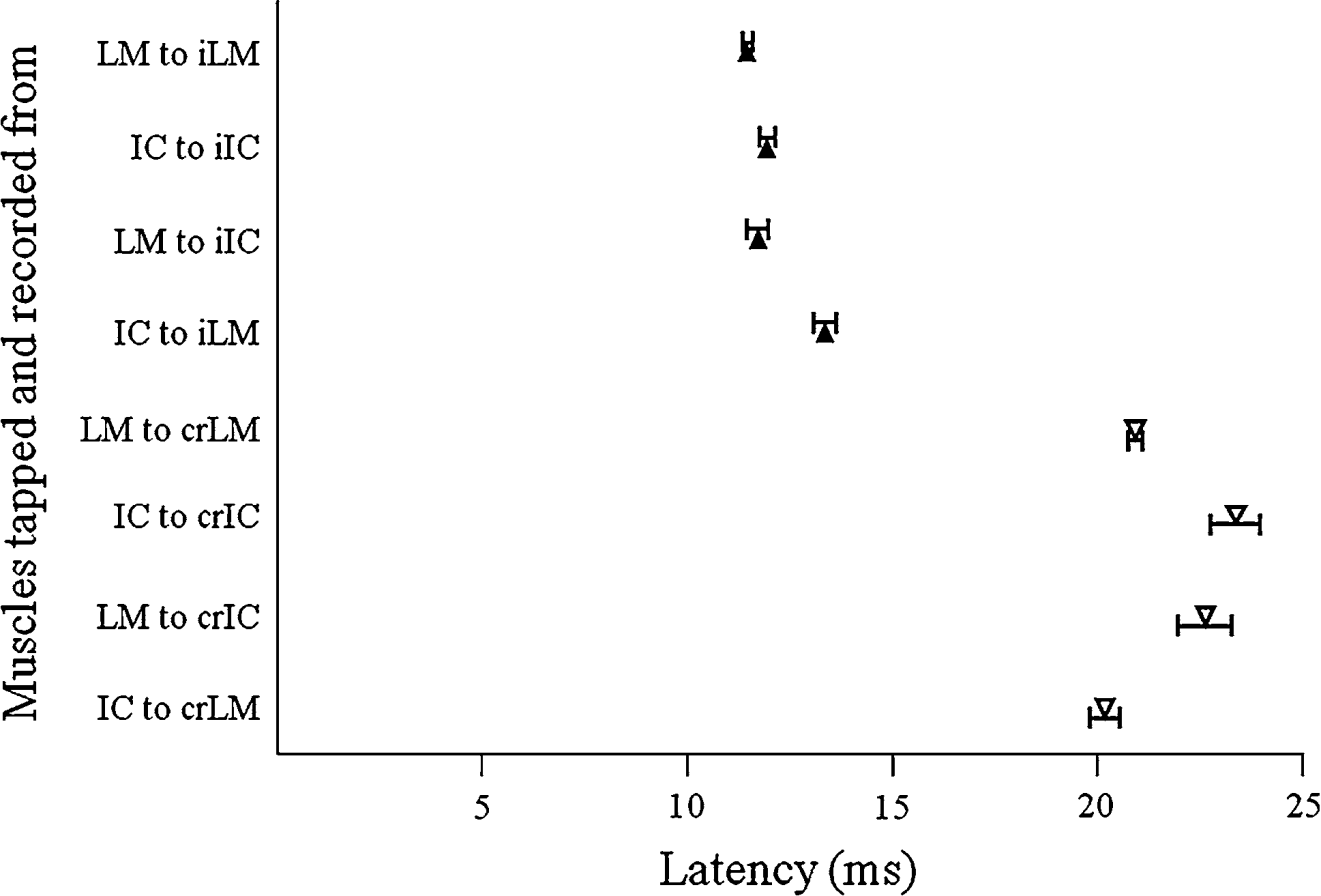
Mean latencies (±SEM) of both excitatory (*filled triangle*) and inhibitory (*open inverted triangle*) short latency reflexes within and between the lumbar multifidus and iliocostalis lumborum muscles on both sides of the vertebral column. *LM* lumbar multifidus, *iLM* ipsilateral lumbar multifidus, *crLM* contralateral lumbar multifidus, *IC* iliocostalis lumborum, *iIC* ipsiltateral iliocostalis lumborum, *crIC* contralateral iliocostalis lumborum

Mechanical taps applied to lumbar multifidus and iliocostalis lumborum evoked short latency excitatory reflexes in both muscles homonymously and these were usually obvious on single sweeps. Figure 2 shows the average of 30 sweeps and a series of five single sweep recordings contributing to the average reflex from (a) the lumbar multifidus muscle in response to tapping the same lumbar multifidus muscle and (b) from the iliocostalis lumborum muscle in response to tapping the same iliocostalis lumborum muscle. The average latency of these excitatory responses from all subjects was 11.8 ± 0.2 ms in lumbar multifidus and 12.3 ± 0.3 ms in iliocostalis lumborum (Fig. 3; All data reported are mean ± Standard error of the mean). The difference between these latencies was not significant. Both these responses are similar to those evoked readily from other postural muscles (Beith and Harrison 2004).

### Conduction velocity of the nerve fibres mediating the homonymous lumbar multifidus and iliocostalis lumborum responses

Calculation of the conduction distance of the lumbar multifidus homonymous reflex using the distance measured from skeletons as detailed in the method compared to the average latency of 11.8 ms, and subtracting 1 ms each for central monosynaptic delay and for delay at the neuromuscular junction, gives a conduction velocity of 51 m/s. The conduction velocity calculated using the fastest measured latency (9.0 ms) is 71 m/s.

Calculation of the conduction velocity of the iliocostalis lumborum homonymous reflex using the same method gives a conduction velocity of 40 m/s (however, see "Discussion" below). The conduction velocity calculated using the fastest measured latency (10.2 ms) is 50.6 m/s.

### Short latency ipsilateral heteronymous responses

**Fig. 4:**
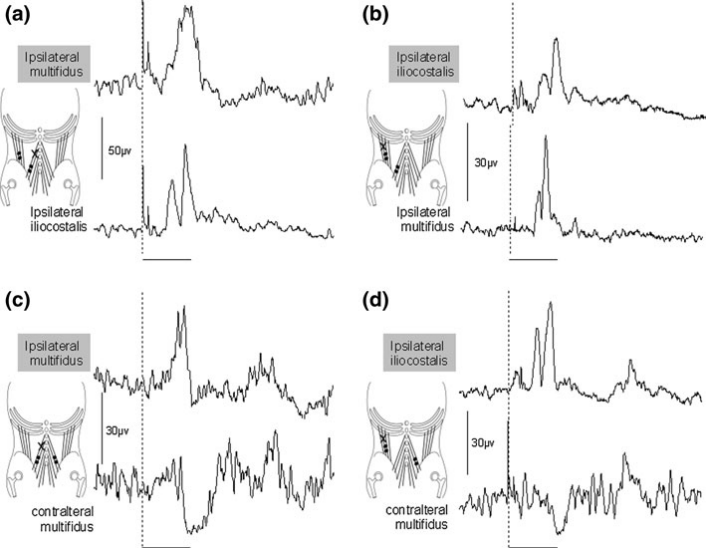
Short latency reflexes in the lumbar multifidus and iliocostalis lumborum muscles when tapping the lumbar multifidus (**a** and **c**) and iliocostalis lumborum (**b** and **d**), the *shaded* title is muscle tapped in each instance. **a**, **b** excitatory homonymous and heteronymous ipsilateral responses in both muscles. **c**, **d** excitatory ipsilateral and inhibitory contralateral responses. The point at which the tap was applied is indicated by the *dashed vertical line*. All responses shown are the rectified then averaged trace of 30 taps. *Horizontal bar*—20 ms

Short latency excitatory ipsilateral heteronymous reflexes were also observed in iliocostalis lumborum when tapping lumbar multifidus and in lumbar multifidus when tapping iliocostalis lumborum (Fig. 4). The latencies of the heteronymous response in iliocostalis lumborum when tapping lumbar multifidus were 11.2 ± 0.4 and in lumbar multifidus when tapping iliocostalis lumborum were 12.5 ± 0.3 (Fig. 3). The latency of all four short latency excitatory reflexes within and between the ipsilateral lumbar multifidus and iliocostalis lumborum muscles were similar (range 11.8–12.5 ms) with no significant difference between them (one-way ANOVA *P* = 0.146).

It could be argued that the responses in muscles other than those tapped but within 10 cm of the muscle tapped were as a result of vibration from the tap evoking homonymous responses. It is however known that the magnitude of vibration when tapping muscles attenuates rapidly (Burke et al. 1983; Beith and Harrison 2004) and the size of stretch reflexes in trunk muscles are proportional to the magnitude of tap applied to them (Kondo et al. 1986). The comparable amplitude of all the excitatory responses in these muscles means it is unlikely that the responses in muscles other than those tapped are purely as a result of vibration of the muscle spindles from within the muscle recorded from.

### Inhibitory responses in contralateral muscles

**Fig. 5:**
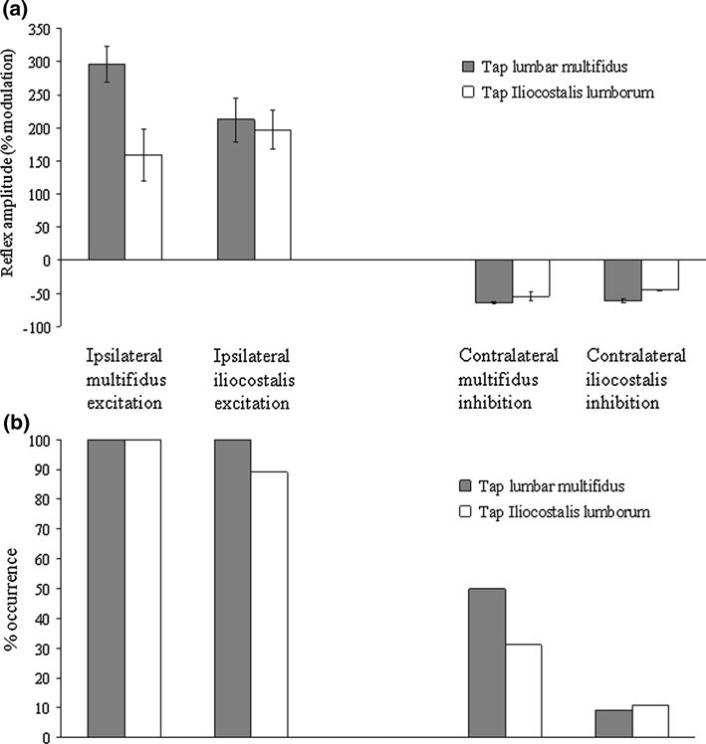
**a** Amplitudes and **b** frequency of occurrence of short latency ipsilateral excitatory and contralateral inhibitory responses

In contrast to the excitatory responses in ipsilateral paraspinal muscles, those observed in the contralateral muscles were inhibitory (Figs. 4, 5). The latencies for the contralateral inhibitory reflexes ranged from 20.9 ± 0.5 to 22.6 ± 1.4 ms (Fig. 3) and were not significantly different from one another (one-way ANOVA *P* = 0.163). On average, the contralateral inhibition occurred 9.1 ms after the excitation in the ipsilateral muscle.

### Reflex amplitude and frequency

The relative amplitudes of the short latency excitatory and inhibitory reflexes are shown in Fig. 5 with the main finding being that the excitatory responses were much easier to evoke than the inhibitory responses. The excitatory response in lumbar multifidus when tapping lumbar multifidus is significantly larger than the excitatory responses from iliocostalis to iliocostalis (*P* = 0.032 post hoc Tukey’s test) and iliocostalis to lumbar multifidus (*P* = 0.017 post hoc Tukey’s test). The inhibitory responses were not significantly different in size from one another.

## Discussion

The present study has identified and characterised the short latency tap-evoked reflexes within and between the paraspinal muscles on both sides of the lumbar spine including homonymous and heteronymous excitatory ipsilateral responses and inhibitory responses in the contralateral muscles.

### Pathways underlying the identified responses

The homonymous reflexes seen in lumbar multifidus and iliocostalis lumborum, in response to tapping each muscle in turn, are similar to those identified in the human abdomen (Beith and Harrison 2004) and build upon our knowledge of reflexes in the large hindlimb muscles in the cat (Eccles and Lundberg 1958; Eccles et al. 1957; Laporte and Lloyd 1952; Liddell and Sherrington 1924). The responses were also easy to evoke being apparent on individual sweeps, which is similar to the stretch reflexes we observed in the abdominal muscles (Beith and Harrison 2004). As such, these homonymous reflexes in the paraspinal muscles are consistent with strong Ia connectivity associated with postural muscles and confirmed in the abdominal muscles (Beith and Harrison 2004 and for a fuller discussion of the relationship between short latency stretch reflexes and postural muscles). Such tap-evoked reflexes therefore seem most likely to be mediated via Ia muscle afferents, at least the shortest latency components, though a contribution from Group II muscle afferents or cutaneous afferents cannot be completely ruled out.

The conduction velocities calculated from the latency of the lumbar multifidus homonymous responses and the measured pathway on human skeletons are also compatible with the stretch reflex responses in the human abdominal muscles (range: 49–52 m/s, Beith and Harrison 2004) and they are compatible with known values for human Ia muscle afferents (Burke et al. 1983).

The conduction velocities calculated from the latency of the iliocostalis lumborum homonymous responses and the measured pathway on human skeletons give values lower than for lumbar multifidus and for the abdominal muscles (range: 49–52 m/s, Beith and Harrison 2004). It may be that the iliocostalis lumborum truly has a slower conduction velocity than the other trunk muscles measured. It may also be that the surface electrodes are further away from the end plate than for the experimental set-up for lumbar multifidus. If so, at least some of the extra time is accounted for by the action potential travelling a greater distance from the end plate to the electrodes. However, if it were assumed that the nerve supply came via one vertebral level lower (L2), this would add an extra 30 mm to the pathway giving an average conduction velocity of 48 m/s and a fastest conduction velocity of 60.5 m/s. Both these values are comparable to the conduction velocities measured from homonymous stretch reflexes evoked in lumbar multifidus and rectus abdominis, external oblique and internal oblique (Beith and Harrison 2004). In addition, the short time span of the rise and fall of the tap used in this study (see "Method") is more consistent with evoking Ia afferent activity from the muscle tapped and not from slower conducting group II muscle afferents. It therefore seems likely that the reflex evoked in iliocostalis lumborum is evoked from Ia afferents within the muscle.

The heteronymous responses observed ipsilaterally in lumbar multifidus when tapping iliocostalis lumborum and in iliocostalis lumborum when tapping lumbar multifidus are also similar to those observed in the human abdomen (Beith and Harrison 2004) and the cat hindlimb (Eccles and Lundberg 1958; Eccles et al. 1957). As the latencies of these responses were similar to those evoked homonymously (non-significant difference between the latencies of homonymous and heteronymous reflexes), it seems likely that these responses are also mediated via Ia muscle afferents.

In contrast to the short latency excitatory responses seen in ipsilateral muscles, responses in the contralateral muscles were inhibitory in nature and less frequently observed, rarely in iliocostalis lumborum, Further, the inhibitory nature of the crossed reflexes between the paraspinal muscles is in contrast to the excitatory nature of the crossed short latency reflexes seen between other trunk muscles in both the abdomen (Beith and Harrison 2004) and the shoulder girdle (Alexander and Harrison 2002). The question therefore remains why the reflex connections between the paraspinal muscles are not similarly excitatory?

### Contrasting contralateral reflex pathways in trunk different muscles

Various studies have investigated the activation patterns of abdominal and paraspinal muscles (i.e. Peach et al. 1998) but the work of Zedka et al. (1998) illustrates the contrast between the function of these two muscle groups most clearly and suggest a possible role for the crossed inhibitory reflex. Subjects sat on a platform that was perturbed in one of three planes, from front to back, side to side and in rotation, whilst activity in abdominal and paraspinal muscles on both sides was recorded using surface EMG. Whilst the left and right paraspinal muscles were co-activated during forward and backward motion, they reacted phasically as agonist and antagonist during left–right perturbation. This latter activation pattern in response to overt stretch of the paraspinal muscle on one side is complimented by the crossed inhibitory short latency reflex between these two muscles. In contrast, irrespective of the direction of the perturbation, the abdominal muscles on left and right sides were always co-activated. Such contrasting patterns of activation compliment the different sign of the heteronymous, crossed reflex pathways observed in both studies, and fit with the anatomical arrangement of the different muscles groups (for a fuller discussion of the role of abdominal stretch reflexes, see Beith and Harrison 2004).

The frequency with which the crossed reflexes (or any reflex) occur for a given stimulus may also reflect their ability to influence motoneurone output. The crossed inhibitory responses between the paraspinal muscles observed here were apparent in less than 50% of all experiments (rarely in iliocostalis lumborum, Fig. 5b), whereas the crossed excitatory responses in the abdominal muscles (Beith and Harrison 2004) and indeed between the trapezius muscles (Alexander and Harrison 2002) are almost always present. The greater difficulty with which the crossed inhibitory paraspinal reflexes are elicited may indicate a reduced ability to influence motoneurone output in the homonymous contralateral muscle, whereas the consistent observation of crossed excitatory responses in the abdominal muscles (Beith and Harrison 2004) and trapezii (Alexander and Harrison 2002) may indicate a greater ability to influence motoneurone output in their respective contralateral homologous muscles.

As the paraspinal muscles exhibit more diverse activation patterns when reacting to different challenges and during different tasks, this may necessitate greater supraspinal control. The greater difficulty in evoking these crossed responses may therefore reflect an appropriate reduction in the influence of these pathways on motoneurone output in these specific muscle groups. As the homologous abdominal muscles are co-activated during the majority of voluntary movements of the trunk (Peach et al. 1998; Zedka et al. 1998) and the left and right trapezius muscles are usually co-activated during various tasks (Alexander and Harrison 2002), the ease with which these excitatory short latency (spinally mediated) crossed reflexes are elicited may indicate their increased ability to affect motoneurone output. As yet there is no direct evidence to support this.

### Crossed inhibitory pathway across the spinal cord

One remaining unexplained aspect of these results is the relatively long difference of 9.1 ms between the onset of the ipsilateral homonymous excitatory response and the crossed inhibition in the homologous paraspinal muscles to the same stimulus, similar to that observed by Zedka et al. (1999) in the erector spinae. Such a delay suggests that the pathway is not the classic disynaptic pathway of reciprocal inhibition via Ia inhibitory interneurons, but may involve a greater number of interneurons, possibly of propriospinal origin.

In summary, tapping the paraspinal muscles produces short latency excitatory reflexes in both the homonymous and heteronymous paraspinal muscles on the same side of the trunk. In contrast, such a tap evokes an inhibitory response in the paraspinal muscles on the opposite side of the trunk though this was observed less than 50% of the time. This is in contrast to the crossed excitatory short latency reflexes, easily evoked and large, seen in the contralateral abdominal muscles and the easily evoked crossed excitation between the trapezius muscles. Such diversity in the arrangement of crossed reflex pathways seems to compliment the most common activation patterns of these muscle groups. As such, the less frequent occurrence of the crossed inhibition in the paraspinal muscles may be due to the complex and variable activation patterns of these muscles, and as such, spinally mediated heteronymous crossed reflex control may be less important in the control of the left and right paraspinal muscles.

## References

[CR1] Alexander CM, Harrison PJ (2002). The bilateral reflex control of the trapezius muscle in humans. Exp Brain Res.

[CR2] Askar OM (1977). Surgical anatomy of the aponeurotic expansions of the anterior abdominal wall. Ann R Coll Surg Engl.

[CR3] Beith ID, Harrison PJ (2004). Stretch reflexes in human abdominal muscles. Exp Brain Res.

[CR4] Burke D, Gandevia SC, McKeon B (1983). The afferent volleys responsible for spinal proprioceptive reflexes in man. J Physiol.

[CR5] Carpenter MB (1985). Neuroanatomy.

[CR6] De Foa JL, Forrest W, Biedermann HJ (1989). Muscle fibre direction of longissimus, iliocostalis and multifidus: landmark-derived reference lines. J Anat.

[CR7] Dimitrijevic MR, Gregoric MR, Sherwood AM, Spencer WA (1980). Reflex responses of paraspinal muscles to tapping. J Neurol Neurosurg Psych.

[CR8] Eccles RM, Lundberg A (1958). Integrative pattern of 1a synaptic actions on motoneurones of hip and knee muscles. J Physiol.

[CR9] Eccles JC, Eccles RM, Lundberg A (1957). The convergence of monosynaptic excitatory afferent on to many different species of alpha motoneurones. J Physiol.

[CR10] Evans AL, Harrison LM, Stephens JA (1991). Cutaneomuscular reflexes recorded from the first dorsal interosseous muscle of children with cerebral palsy. Dev Med Child Neurol.

[CR11] Fuglevand AJ, Winter DA, Patla AE, Stashuk D (1992). Detection of motor unit action potentials with surface electrodes: influence of electrode size and spacing. Biol Cybern.

[CR12] Katz B, Miledi R (1965). The measurement of synaptic delay, and the time course of acetylcholine release at the neuromuscular junction. Proc R Soc Lond B.

[CR13] Kondo T, Bishop B, Shaw CF (1986). Phasic stretch reflex of the abdominal muscles. Exp Neurol.

[CR14] Laporte Y, Lloyd DPC (1952). Nature and significance of the reflex connections established by large afferent fibres of muscular origin. Am J Physiol.

[CR15] Liddell EGT, Sherrington CS (1924) Reflexes in response to stretch (Myotatic Reflexes). Proc Royal Soc Lond (B) Biol Sci 96:212–242

[CR16] Macintosh JE, Valencia FP, Bogduk N, Munro RR (1986). The morphology of the human lumbar multifidus. Clin Biomech.

[CR17] Matthews PBC (1972). Mammalian muscle receptors and their central actions.

[CR18] Myriknas SE, Beith ID, Harrison PJ (2000). Stretch reflexes in the rectus abdominis muscle in man. Exp Physiol.

[CR19] Peach JP, Sutarno CG, McGill SM (1998). Three-dimensional kinematics and trunk muscle myoelectric activity in the young lumbar spine: a database. Arch Phys Med Rehabil.

[CR20] Rizk NN (1980). A new description of the anterior abdominal wall in man and mammals. J Anat.

[CR21] Shindo H (1995). Anatomical study of the lumbar multifidus muscle and its innervation in human adults and fetuses. Nippon Ika Daigaku Zasshi.

[CR22] Standring S, Healy J, Johnson D, Williams A (2004). Gray’s anatomy: the anatomical basis of clinical practice.

[CR23] Stein RB, Estabrooks KL, McGie S, Roth MJ, Jones KE (2007). Quantifying the effects of voluntary contraction and inter-stimulus interval on the human soleus H-reflex. Exp Brain Res.

[CR24] Tani T, Yamamoto H, Ichimiya M, Kimura J (1997). Reflexes evoked in human erector spinae muscles by tapping during voluntary activity. Electroencephalogr Clin Neurophysiol.

[CR25] Van Boxtel A (1986). Differential effects of low-frequency depression, vibration-induced inhibition, and posttetanic potentiation on H-reflexes and tendon jerks in the human soleus muscle. J Neurophysiol.

[CR26] Wohlert AB (1996). Reflex responses of lip muscles in young and older women. J Speech Hear Res.

[CR27] Wood JL, Smith A (1992). Cutaneous oral-motor reflexes of children with normal and disordered speech. Dev Med Child Neurol.

[CR28] Zedka M, Kumar S, Narayan Y (1998). Electromyographic response of the trunk muscles to postural perturbation in sitting subjects. J Electromyogr Kinesiol.

[CR29] Zedka M, Prochazka A, Knight B, Gillard D, Gauthier M (1999). Voluntary and reflex control of human back muscles during induced pain. J Physiol.

